# “10,000 Available” or “10% Remaining”: The Impact of Scarcity Framing on Ticket Availability Perceptions in the Secondary Ticket Market

**DOI:** 10.3390/bs13040338

**Published:** 2023-04-18

**Authors:** Wonsok (Frank) Jee, Moonsup Hyun

**Affiliations:** 1 School of Marketing, Entrepreneurship, Sport Management, Hospitality, and Tourism Management, Western Carolina University, Cullowhee, NC 28723, USA; 2 Department of Business and Economics, Utica University, Utica, NY 13502, USA

**Keywords:** pricing, scarcity messages, framing, numeracy, secondary ticket market

## Abstract

The aim of this study was to investigate the effect of numeracy framing and demand on participants’ perceived ticket availability and likelihood of finding a lower-priced deal in the secondary ticket market for National Football League (NFL) games. A total of 640 participants were recruited via Qualtrics where participants were solicited electronically via 10 date-specific email blasts prior to a New York Giants Sunday Night Football home game. Participants were randomly assigned to one of five treatment conditions (control, percentage frame × low demand, percentage frame × high demand, frequency frame × low demand, frequency frame × high demand) to complete an online survey. Multivariate analysis of variance (MANOVA) was performed to discern overall differences in the mean likelihood scores of the dependent variable between groups. The results showed that participants presented with the “percentage” frame perceived tickets as less available than those presented with the “frequency scarcity” frame, and the effect was greater for high-demand games. Additionally, game demand moderated the effect of scarcity framing on participants’ perceived ticket availability and expected lower rate. Several manipulation checks were applied to ensure the study’s validity. The findings of this study have practical implications for ticket marketers in the sport industry to effectively frame scarcity information and facilitate transactions for online buyers and sellers.

## 1. Introduction

The secondary ticket market for sport, music, and entertainment (i.e., the ticket resale marketplace) mushroomed into a USD 15 billion industry in 2021 and is expected to balloon to USD 68 billion by 2025 [1,2]. Roughly 1500 ticket service companies, including ticket resale platforms, were conducting business as of 2015 [3]. Mergers and acquisitions have increased industry concentration. StubHub is the largest player in this space and was initially acquired by eBay in 2007 for USD 310 million. The platform was later sold to Swiss ticket vender Viagogo for a whopping USD 405 billion [4]. The secondary ticket market has grown considerably with the emergence of Internet-based platforms that enable buyers and sellers to engage in transactions worldwide [5]. As the global mobile apps market expands, many service providers are developing mobile applications to offer consumers simple access. The virtual ticket marketplace has hence become fiercely competitive: vendors are vying to provide the ultimate user interface (UI) design. The goal of UI design is to create an interface through which users can easily, efficiently, and enjoyably interact with a product. StubHub’s early success and subsequent rise to the top of the secondary market has been attributed to the site’s simple yet informative UI. In recent years, there has been increased competition in the secondary ticket market in the sport industry. To make transactions more appealing for online buyers and sellers, ticket marketers are focusing on standout user interface design on ticket distribution platforms. They are experimenting with ways to effectively frame scarcity information, incite urgency among buyers, and facilitate transactions.

Consumers’ penchant to visit the secondary marketplace to buy tickets, coupled with high competition among providers, has compelled ticket platforms to conduct a/b testing. Companies have also experimented with marketing techniques in the digital space to distinguish themselves and make their platforms more appealing to buyers and sellers alike [6,7]. Nonetheless, research to date has merely empirically assessed the effectiveness of emerging practices in the virtual secondary ticket market; the practices themselves have yet to be tested (Brett Goldberg, CEO and co-founder of TickPick, personal communication, September 2017). As a late follower and startup founded in 2011, TickPick was built on a business model focusing on buyers’ disdain for hidden fees. The platform instead quotes all-inclusive single prices that are guaranteed to be less at the purchase stage. This approach directly contradicts the market leader StubHub’s business model where a low price is quoted in an initial search, but taxes and commission fee surcharges are added to the final price. StubHub believes that consumers will only remember the price anchor (i.e., the first piece of information) and forget the total price paid. Thanks to a standout UI and effective marketing strategies, TickPick was ranked the fourth largest secondary market in 2021 [1]. This platform, along with RazorGator, SeatGeek, TicketIQ, and Vivid Seats, has found success in this competitive industry. Meanwhile, many other players have perished. Determining what does and does not work (e.g., via a/b testing) seems essential to thriving in this space—as does being able to understand sport fans and buyers’ decision-making processes in the ticket market.

Sport fans who book tickets in a dual market of primary and secondary providers must grapple with many decisions. Popp et al. identified seven salient attributes when booking sport event tickets online: (1) ticket purchase timing, (2) seat availability, (3) price fluctuations, (4) fee transparency, (5) number of price points, (6) fraud risk, and (7) price valuation assessment [8]. Official partnerships have recently been stressed to counter fraud risk [5]. Won and Shapiro examined the impacts of fee transparency, price fluctuations, and the number of price points by exploring the role of price bundling on consumers’ attitudes and behavior [9]. The current work focuses on two other major factors—ticket purchase timing and seating availability—to further examine how these aspects influence consumers’ likelihood of booking through the secondary ticket market.

**Figure 1:**
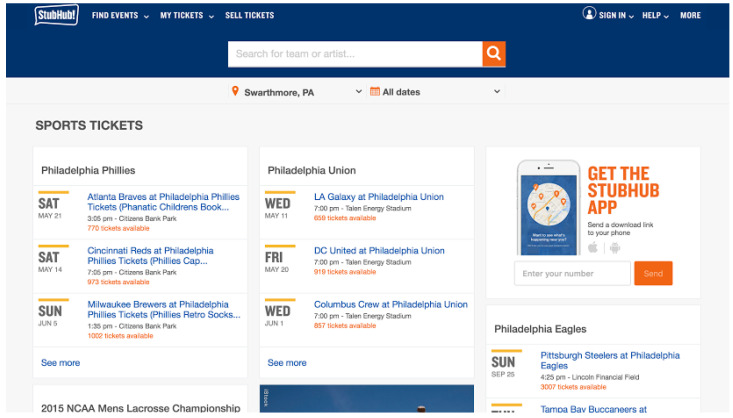
Example of Scarcity Through Frequency Numeracy Framing. Source: StubHub.

Ticket marketplace distributors primarily display visuals and graphics on their websites to convey information on seat availability (see Figure 1). The premise behind this type of information disclosure is to signal scarcity and create a sense of urgency among buyers, thereby encouraging purchases [10]. This priority on scarcity has been augmented through creative fear of missing out (FOMO) marketing initiatives. Specifically, marketers strive to craft messages that appeal to consumers’ desire to seize an opportunity before it slips through their fingers [11].

Typically, when purchasing event tickets online, consumers are exposed to information on product scarcity based on raw numbers (e.g., the total number of tickets available for certain seat sections). The way in which information is presented or framed affects individuals’ purchase-related evaluations and choices [12]. For example, Levin demonstrated more favorable associations for a purchase of ground beef when the beef was described as “80% lean” rather than “20% fat” [13]. In the medical field, symptom evaluation, patient compliance, treatment, and doctor selection have all been shown to feature framing effects [14]. Patients presented with risk information about a medication’s side effects in a percentage format (i.e., 10% of patients experience a blistering rash) perceived medication as less risky than when given risk information in frequency format (10 out of 100 people experience a blistering rash). Frequency formats appear to increase risk perceptions over percentage formats for less numerate participants. The present study tests whether consumers’ scarcity perceptions vary when information about seat availability is presented using different numerical frames (i.e., as frequencies or a percentage).

Marketers can struggle when sellers overestimate demand or when seeking to liquidate low-demand game tickets. Broker data showing tickets selling for pennies on the dollar (or not at all) in the secondary market highlight the need for improved marketing and serious consideration of scarcity framing. For instance, when using different numerical cues to gauge scarcity, are consumers even measuring the same phenomenon? If a stadium’s full capacity is 40,000 seats, will a buyer’s risk perception vary when the information is presented as “400 seats available” compared to “Less than 1% remaining”? Contrarily, when there is relatively low demand for a sport event, does a percentage generate more urgency (e.g., “56%” vs. “22,400 seats remaining”)? Is any numeracy format in fact more effective than another when conveying scarcity? From a marketing perspective, it would be ideal to implement whichever frame signals greater scarcity and evokes a sense of urgency for consumers to book/purchase in an uncertain scenario. As such, this study explores which frame is more useful in signaling higher scarcity perceptions and amplifying consumers’ propensity to book early.

Several research questions guide this work:

RQ_1_: Do consumers’ expectations of ticket availability with respect to an upcoming professional sport event differ over time?

RQ_2_: Do consumers’ expectations of finding lower-priced tickets with respect to an upcoming professional sport event differ over time?

RQ_3_: Which numeracy frame (frequency vs. percentage) of seat availability more effectively signals scarcity with respect to high- and low-demand sport events?

## 2. Theoretical Background

### 2.1. Generic Advanced Decision-Making Model and Sport Ticket Booking Behavior

**Figure 2:**
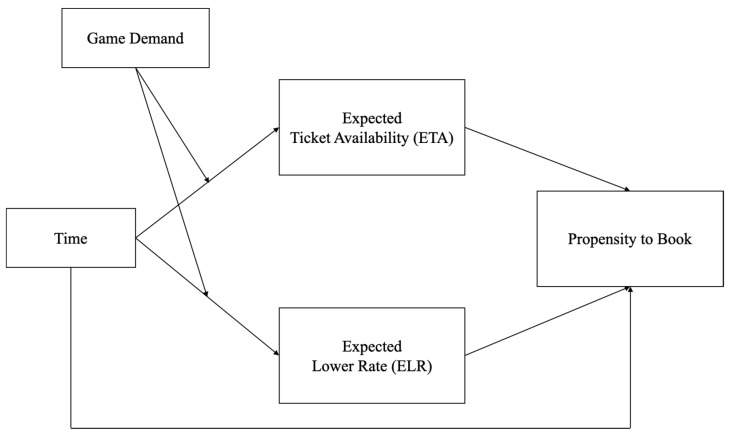
Generic Advanced Decision-Making Model.

To address the above research questions, the theoretical framework of Dwyer, Drayer, and Shapiro’s generic advanced decision-making model (view Figure 2) was adapted in this study [15]. This model consists of two constructs reflecting risk perceptions that dictate consumers’ advance-purchase decisions in relation to sport tickets: expected ticket availability (ETA) and expected lower rate (ELR). Risk perceptions refer to people’s subjective judgments about the characteristics and severity of a risk [16]. Using a 19-day advance-purchase timeframe, Dwyer et al. found that ETA (an indirect measure of sellout risk) and ELR both decreased for a generic sport ticket product category when nearing the day of the event [15]. Consumers’ lower ETA and ELR risk perceptions should ultimately increase the probability of purchasing event tickets.

The fluctuating ETA and ELR patterns revealed in Dwyer et al.’s work underline the need to develop strategies that capture how consumers deal with uncertain information cues in the advance ticket sales market [15]. Risk perceptions are distinct from actual risks, as the former are shaped by a range of affective, cognitive, contextual, and personal factors [17]. This study addresses context by investigating how consumers’ subjective risk assessments (i.e., ETA and ELR) can be further distorted by information presented in the purchase environment. More precisely, under the generic advanced decision-making model, this study tested which numerical frame (frequency vs. percentage) generated greater urgency (i.e., ETA and ELR risk perceptions) that inspired consumers to either book immediately or wait. This research thus aimed to identify when the scarcity problem was most likely to manifest for buyers and to provide guidance on the optimal framing of seat availability messages. 

Accordingly, the following hypotheses were empirically tested:

**H1.**  *Based on the generic advanced decision-making model, we predict that there will be a significant relationship between purchase timing and participants’ ETA and ELR. Specifically, as purchase timing increases, we expect ETA and ELR to decrease and vice versa*.

**H2.**  *After controlling for the effects of purchase timing, we hypothesize that higher game demand will result in lower ETA and ELR, while lower game demand will lead to higher ETA and ELR*.

### 2.2. Framing Effect

A frame refers to a mental model of a decision problem [18]. People use this frame to solve the problem; it includes details about the elements of the problem (i.e., information) as well as the context. The framing literature suggests that decision makers will use information in the context of the frame that is presented to them without attempting to reframe it [19,20]. Although information about a particular problem might remain the same, it can be perceived, organized, and interpreted differently. The problem can also be resolved in varied contexts, by certain people, or at different times. Behavioral researchers have collectively referred to these distinct ways of looking at the same problem as a “different frame” [21,22]. The “framing effect” essentially involves the same problem being presented using different information representations. People have been found to heavily modify, or even reverse, their decisions based on this effect [22].

Tversky and Kahneman’s introduction of the Asia Disease Problem was among the earliest examples of how valence affects one’s willingness to take a risk and, consequently, the malleability of human decision making. Imagine that the United States is preparing for an outbreak of an unusual Asian disease that is expected to kill 600 people, and two programs have been proposed to combat it. Scientific estimates of the consequences of each program are known. The positive frame is as follows: if Program A is adopted, 200 people will be saved; if Program B is adopted, there is a 1/3 probability that all 600 people will be saved and a 2/3 probability that no one will be saved. Under the negative frame, if Program C is adopted, 400 people will die; if Program D is adopted, there is a 1/3 probability that no one will die and a 2/3 probability that all people will die. Because Programs A and C and Programs B and D are logically equivalent, people’s preferences should be identical. Their preferences also should not shift from risk seeking to risk avoidance simply because of how the problem is described. However, Tversky and Kahneman reported that 71% of participants would choose Program A rather than B in the positive frame, while 72% of participants would choose Program D rather than C in the negative frame [22].

A considerable body of work subsequently demonstrated that the framing effect is a robust phenomenon that applies to various decision-making problems [23,24]. Pertinent domains include the economy [25], lifesaving [26], resource allocation [27], and management [28]. Framing effects have also been documented in medical and clinical settings (e.g., decisions made by health care providers and patients), perceptual judgments, consumer choices, responses to social dilemmas, bargaining behavior, auditing evaluations, and many other decision types. 

Kamoen et al. explored whether sports speakers’ and writers’ adjustments to their frame choices in athlete-related messaging affected sport fans’ perceptions of athletes’ performance [29]. In asking participants to rate a tennis player’s previous performance, some participants were informed that the player had won 8 out of the last 10 games (positive frame), whereas others were told that she had lost 2 out of the last 10 games (negative frame). Risk was not an issue in either case, yet participants who were told success rates rather than failure rates rated the player’s performance significantly [29]. These positive and negative descriptions may be truth-conditionally equivalent. Even so, research continues to indicate that the frame choice affects people’s evaluations of the described entity.

### 2.3. Scarcity Framing and Numeracy 

Scarcity is an intriguing concept to study with respect to decisions made in certain conditions. According to the scarcity principle, opportunities seem more valuable when they appear more limited [30]. Scarcity techniques predominantly fall into two categories: suppliers can either restrict the number of products/deals available (e.g., “Only 500 tickets available”) or offer promotions for a limited time (e.g., “Only available for 15 days”). The fundamental principles of these messages differ. Under the quantity condition, suppliers restrict products to a limited number of available units; under the limited-time condition, suppliers encourage consumers to purchase as soon as possible to generate maximum revenue during the promotional period. The former approach creates a sense of urgency in that consumers feel as though they are competing with other buyers because the number of available products declines as others purchase [10]. 

Numeracy is defined as “how facile people are with basic probability and mathematical concepts” and is an important consideration in framing [31]. Individuals’ perceptions of numbers, ratios, and percentages can be explored using numeracy tests, such as the Berlin Numeracy Test [32]. Other assessments emphasize statistical numeracy. Numeracy tests are especially applicable to judgment and decision-making tasks [33]. Black, Woloshin, and Welch discovered that numeracy was strongly related to understanding the benefits of mammography, which has implications for the framing of numbers [34]. In another study in medicine, Peters, Sol Hart, and Fraenkel found that numeracy influences risk perceptions when different information frames and number formats are used to present medication risks [35]. 

This study attempts to replicate framing effects in the context of consumers’ risk perceptions and booking behavior in the secondary sport ticket market. The researchers took the same information and presented it differently to two groups of participants and then compared participants’ decisions. In some cases, the framing manipulation had no effect; when an effect was observed, the typical pattern involved either a choice reversal or a choice shift in the direction of lower willingness to take a risk when choices were framed positively than when choices were framed negatively. The authors theorized that participants’ perceptions of scarcity would be influenced by the frame of the number of seats available. When framed differently under high (negative) or low demand (positive), a choice reversal was expected to occur [12].

Accordingly, the following hypotheses were empirically tested:

**H3.**  *After controlling for the effects of purchase timing, we anticipate that seat availability framed through percentages will signal greater scarcity compared to frequency framing. Hence, we predict that participants presented with seat availability framed through percentages will have lower ETA and ELR than those presented with frequency framing*.

**H4.**  *We further hypothesize that game demand will moderate the effect of numeracy framing. Specifically, we expect a choice reversal between high- and low-demand games. For high-demand games, we predict that frequency framing will result in lower ETA and ELR than percentage framing, while for low-demand games, we predict that percentage framing will result in lower ETA and ELR than frequency framing*.

## 3. Methods and Measurements

### 3.1. Sample and Procedures

**Figure 3:**
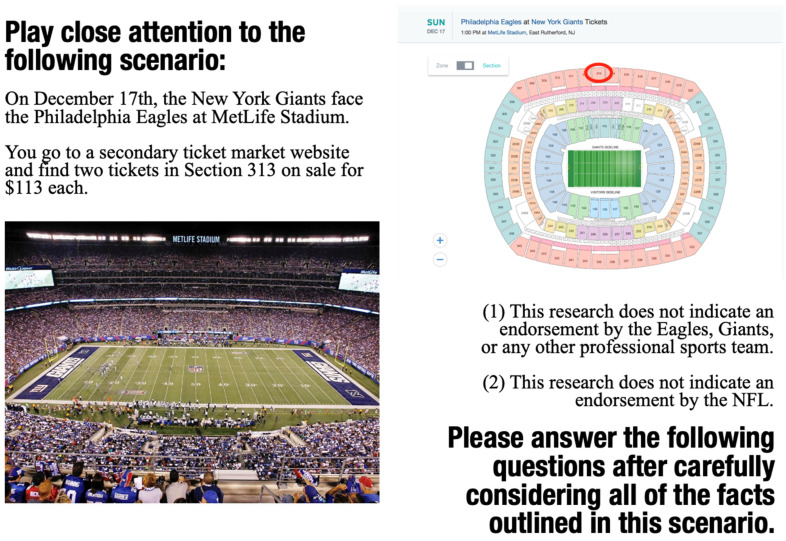
Vignette of Ticket Purchase Scenario.

A total of 640 participants were recruited via Qualtrics, a consumer insight panel, to complete an online survey. Participants were solicited electronically via 10 date-specific email blasts and recruited prior to a New York Giants home game on Sunday Night 17 December 2017. Participants were asked to complete two screening questions to verify that they met eligibility criteria (i.e., New York- and Philadelphia-area fans). Participants who agreed to take part in the study were next randomly assigned to a vignette ticket purchase scenario. The main study used a 2 (demand: high vs. low) × 2 (numeracy framing: frequency vs. percentage) factorial between-group design; participants were randomly assigned into five groups, including a control-group scenario. The hypothetical scenario controlled extraneous factors of seat location, time, and event date (see Figure 3). According to the scenario, participants were instructed to buy two tickets prior to the event. They were asked a pair of questions that estimated the likelihood of future events (i.e., anticipated ticket availability and the expected likelihood of finding a lower-priced deal later). These questions were followed by several demographic items addressing participants’ attitudes and behavior, including their ticket purchase experience, knowledge, and fan involvement. 

A between-subject design was adopted because participants were only exposed to one condition. This approach enabled the authors to ensure that participants’ scores were not influenced by factors such as sensitization or realization of what was being measured, thereby increasing the possibility of eliciting sound tendencies. Participants also did not experience order effects, such as the carryover of perceived status. Systematic differences between groups were therefore minimized or eliminated in order to achieve group equivalence. 

### 3.2. Manipulation Check 

Challenges to online survey data collection include self-misrepresentation, inattention to survey responses, and high attrition rates [36]. Several manipulation checks and screening questions were applied to uphold this study’s validity. The first screening question addressed self-misrepresentation by verifying whether the participant was an actual fan of the team. Along with the geographical screening code, it was imperative to confine the sample to Giants home fans for the purpose of this study. The screening question contained a photo and asked participants to identify an all-pro wide receiver on the New York Giants. A memory recall attention check question appeared immediately after participants read the scenario description: they were asked to recall the exact price quoted in the purchase scenario and were not given the option to return to that page. Participants who responded incorrectly were directed out of the study. Attention check questions were also included in Likert-scale matrices throughout the survey. To check whether participants were paying attention as they completed the study, they were sporadically asked to either click “strongly agree” or “strongly disagree.” Participants who completed the survey excessively quickly (i.e., in less than 5 min) or whose surveys were incomplete were also eliminated from data analysis.

### 3.3. Dependent Variable

The two dependent variables for this study were ETA (i.e., participants’ assessment of expected ticket availability) and ELR (i.e., participants’ perceived likelihood of finding a similar or lower-price ticket any time before the scenario date and the actual game [15]). The prompts accompanying each of these variables were as follows:

Expected ticket availability (ETA)

I believe the chance the same or very similar tickets will be available between tomorrow (DATE) and today (DATE) is __________%. 

(Please indicate a number between 0 and 100)

Expected likelihood of finding a better deal (ELR) 

I believe the chance that I could find the same or very similar tickets somewhere else at a price lower than $(PRICE) each between tomorrow (DATE) and today (DATE) is ______%. (Please indicate a number between 0 and 100)

### 3.4. Independent Variable

The independent variables were directly manipulated through vignette descriptions. Content analysis was conducted to determine the level of high demand and low demand for National Football League (NFL) games. The highest NY Giants attendance figure for the NFL season was in Week 7 (78,527 fans), while the lowest was in Week 10 (73,210 fans). The total capacity of seats at MetLife stadium is 82,500. Therefore, the high-demand condition was demonstrated through either the message “825 tickets remaining” (frequency) or “1% remaining” (percentage). Information in the low-demand condition was represented through either “16,500 seats remaining” (frequency) or “20% remaining” (percentage).

### 3.5. Statistical Analysis

**Figure 4:**
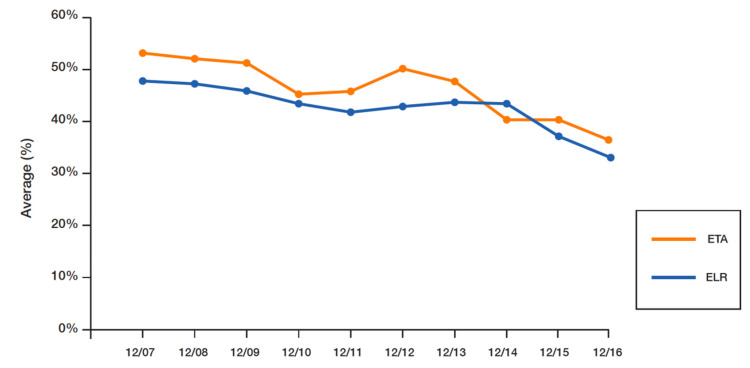
Graphical Trends in ETA and ELR Estimates by Days Leading up to the Event.

All statistical analyses were performed in R 3.6.2 and SPSS 27. A total of 640 New York area sports fans responded to the email solicitation. Table 1 lists demographic information for the sample. Table 2 displays the number of observations corresponding to the 10 time-level treatments as well as the averages and standard deviations for each dependent variable (ETA and ELR). Perceived ticket availability was highest 10 days prior to the event (53.72%) and lowest the day before the event (37.89%). The same pattern was observed for ELR: it was highest (48.92%) 10 days prior to the event and dropped significantly to 35.88% a day before the event. In general, as the event date drew closer, participants’ perceived probability of ticket availability and the likelihood of finding a lower-priced ticket declined significantly (view Figure 4).

Multivariate analysis of variance (MANOVA) was performed to discern overall differences in the mean likelihood scores of the dependent variable between groups. MANOVA is appropriate when multiple dependent variables are moderately correlated [37]. Two 2 × 2 factorial analyses of variance (ANOVAs) were carried out to identify differences in the mean assessments for each treatment. The main effect results were then analyzed to determine whether the structural framing of numeracy scarcity and price anchoring affected participants’ probability estimations of uncertainty (i.e., ETA and ELR). Tukey’s post hoc tests were further conducted to detect which combined treatment level generated significant differences compared with other groups. Additionally, because the same dependent variables were used in two separate procedures, a Bonferroni adjustment was made. The significance value was set at 0.025 to account for all main effects.

## 4. Results

Based on Wilks’ statistic, the MANOVA revealed a significant effect of time period on participants’ ETA and ELR assessments (Λ = 0.954, *F*(18, 1262) = 1.653, *p* < 0.05). The descriptive statistics for the dependent variables are reported in Table 2. The effect size, measured using partial η^2^, was 0.023.

Table 3 provides the main effect results for game demand and scarcity framing. The main effects of scarcity framing on ETA and ELR (per a factorial ANOVA) showed statistically significant differences (ETA: *F*(1420) = 19.040, *p* < 0.001; ELR: *F*(1420) = 21.418, *p* < 0.001). The proportion of participants provided with the “raw numbers” frame (52.13%, *n* = 213) who felt that the same or similar tickets would be available between the scenario date and the game day exceeded the proportion of participants provided with the “percentage” frame (40.63%, *n* = 208). Meanwhile, more participants given the “frequency” frame (50.38%, *n* = 213) expected to find a similar or lower-priced deal versus participants in the “percentage” condition (38.33%, *n* = 208).

A factorial ANOVA of the main effects of game demand on ETA and ELR again unveiled significant differences (ETA: *F*(1420) = 5.359, *p* < 0.05; ELR: *F*(1420) = 2.672, *p* < 0.1). On average, the proportion of participants shown the “low” game demand scenario (51.24%, *n =* 203) who perceived the same probability that identical or similar tickets would be available between the scenario date and the game day exceeded that of participants shown the “high” game demand scenario (42.08%, *n* = 218). Participants in the “low” game demand scenario (46.61%, *n* = 203) expected the probability of finding a similar or lower-priced deal to be higher than those in the “percentage” condition (37.14%*, n =* 218).

The interaction effect was statistically significant in terms of the moderating impacts of game demand and scarcity framing on participants’ ETA and ELR (ETA: *F*(1420) = 0.142, *p* < 0.01; ELR: *F*(1420) = 0.036, *p* < 0.01). In the high-demand scenario, the frequency frame was associated with lower ETA (37.89%; ELR: 35.64%, *n* = 106) than the percentage frame (ETA: 49.03%; ELR: 49%, *n* = 112). The opposite effect applied in the low-demand scenario, where the percentage frame led to lower ETA (43.65%; ELR: 41.26%, *n* = 101) compared with the frequency frame (ETA: 55.40%; ELR: 51.84%, *n* = 102).

## 5. Discussion

This study investigated the impacts of purchase timing and the framing effects of scarcity in the advanced-booking setting of a professional sport event. Event game demand was taken as a potential moderator between scarcity framing and consumers’ estimations of ticket availability and finding lower-priced deals. Results suggest that, as the amount of time before the event decreased, participants’ estimations regarding ticket availability and finding a lower price declined significantly. In addition, participants who were presented with a high-demand game scenario had lower estimations of ticket availability and the likelihood of finding a lower price overall than those given the low-demand scenario. Participants in the latter condition who were presented with the percentage frame also had lower ETA and ELR risk estimations than participants given the frequency frame. Contrarily, respondents in the high-demand game scenario who were exposed to the frequency frame displayed lower risk estimations for ETA and ELR.

### 5.1. Theoretical Implications

With respect to theoretically extending the generic advanced decision-making model, Dwyer et al. called for future sport marketing research to include data from multiple time points to determine why consumers’ perceptions of ticket prices and availability change [15]. This study directly responded to this recommendation by collecting data across a 10-day time frame and testing the influence of purchase timing on consumers’ perceived risk in an advance ticket purchase environment. In addition, the distinct impacts of scarcity framing and observed choice reversal on consumers’ perceptions of price and availability offer novel empirical insight to the advance ticket purchase literature. Numeracy framing effects have been replicated in multiple disciplines. However, these consequences have received thin coverage in the sport context. This study revealed statistically significant differences in participants’ estimations via seat availability framing.

Several possible explanations exist for this choice reversal phenomenon via numeracy framing. Perhaps sport fans generally do not prioritize numeracy and thus opt not to closely consider total seat capacity when forming scarcity perceptions about high-demand games. Alternatively, when game demand and risk are both low, seat availability provided as a percentage may simply serve as more information to guide fans’ decisions. Choice reversal could also be a function of the number-size framing effect; for example, people are more sensitive to the change from 1 to 2 than to the change from 101 to 102. Although the size of this change is the same (i.e., an increase of one), larger numerical percentages framed on a scale from 0 to 100 may elicit more sensitivity than frequencies expressed from 0 to 100,000. These conjectures are beyond the scope of this work and require further study. It is nevertheless important to note that the order of risk perceptions was reversed based on expected game demand. Are these results unique to the contexts of sport tickets and the secondary ticket market? Replication with different product types and industry domains is strongly suggested to answer this question.

### 5.2. Practical Implications

Practically speaking, the results related to time and consumers’ advance purchases in the secondary ticket market can offer an auction type simulation that may be useful for teams implementing dynamic ticket pricing in the primary market. Drayer found that primary market ticket prices gradually increased as the game drew closer; in the secondary ticket market, prices rose initially (approximately a month before the game) and dropped considerably leading up to game time [7]. Sport organizations might apply these tactics to protect the integrity of their ticket prices and encourage advance sales. At the same time, this strategy effectively ignores consumers’ expected valuation of the ticket market over time as seen in the present study. The sport industry is moving toward customer-focused pricing rather than previously popular markup pricing to cover organizational costs [7]. Sport marketers must continue to balance traditional ticket sales strategies with an understanding of consumers’ responses to ticket-purchase stimuli in the digital box office environment.

Moreover, sport marketers should vary and optimize seat availability messages (e.g., based on high vs. low demand) to amplify buyers’ sense of urgency and capitalize on FOMO marketing opportunities in digital environments. As mentioned in the introduction, sport ticket purchases have shifted to mobile settings. UI has thus become a prime factor in businesses’ success. Companies such as Gametime have oriented themselves nearly entirely around mobile-based ticketing for millennials. For many teams, leagues, and ticket sellers, the millennial audience represents from one-quarter to one-third of their overall revenue base. According to Ticketmaster’s study on “Ticket Buying Among Millennials” (constituting Ticketmaster’s largest customer segment and one-third of its event database), these consumers are 40% more likely to turn to another website for tickets [38]. They are also 26% more likely than other consumer groups to use the secondary market. Individuals are irrational decision makers, including when deliberating on sport ticket purchases: consumers do not have infinite time to search for the best deal and compare prices. Many systemized decisional biases exist beyond framing effects as well. These biases concern price anchoring [39] and reference pricing [40] along with time pressure and asymmetric dominance [41]. Secondary ticket market platforms can further test and strategically implement these options in their marketplaces as appropriate.

### 5.3. Limitations and Future Research

Although this study offers several practical implications, limitations should be noted. First, the experiments featured a Sunday Night game with a 7 PM ET kickoff time and a competitive NFC East divisional rivalry (NY Giants vs. Philadelphia Eagles). To demonstrate framing effects, it was essential to tightly control the potential impacts of extraneous variables to best focus on the proposed relationships. Yet, multiple questions remain. To what extent might these game attributes affect the experimental results? For example, would findings have differed for a single-header Monday, Thursday, or Sunday Night Football game? What if the teams were less popular? Gierl, Plantsch, and Schweidler did not find significant variation between famous brands and fictitious generic brands in their quasi-experimental study [42]. However, researchers should explore how seat location, featured primetime scheduling, and consumers’ brand attachment to a specific team may moderate the levels of risk perceptions on ticket availability and lower prices.

Another potential limitation of this study entailed the NFL context. Fans have the opportunity to attend a set number of games per season: each team hosts 10 home games for 17 weeks. Compared with the National Basketball Association (41 home games for 19 weeks) or Major League Baseball (81 home games for 26.5 weeks), the baseline scarcity for each NFL home game is higher. Game attendance tends to be high for NFL games, hence why ETA and ELR measures are higher than for other sport events. Scholars should compare the impact of time on ticket purchase risk perceptions across professional sport leagues to identify contextual differences.

Lastly, the study sample consisted solely of New York and Philadelphia area sport fans to understand their decision-making processes and booking behavior. As avid devotees to their home team, fans are primarily motivated by FOMO around being able to support their favorite team and players. Controlled sampling (e.g., by eliminating non-fan consumer segments) clarifies the role of context on the variables under examination but tempers results’ generalizability. Actively engaged players also exist in the secondary market, chiefly casual sales prospects (i.e., people who and who are not avid fans but instead see a ticket purchase as a profit-earning opportunity). Decision agents driven by disparate objectives will likely evaluate risk (ETA and ELR) uniquely in the advance-purchase environment. Subsequent work should analyze how the generic advanced decision-making model may fit with these contrasting consumer segments.

## Figures and Tables

**Table 1:** Sample Demographics.

**Age**	33.674, Mean	**Ethnicity**	76.3%, Caucasian
	11.792, St. Dev		12.3%, Hispanic
**Gender**	69.1%, Male		6.1%, Asian
	30.9%, Female		5.3%, Other
	12.5%, Less than USD 20 K		21.5%, High school
**Household Income**	20.7%, USD 20 K–USD 40 K	**Education**	4.1%, Bachelor’s degree
	22.9%, USD 40 K–USD 60 K		18.0%, Graduate degree
	24.7%, USD 60 K–USD 80 K		16.5%, Professional degree
	15.0%, USD 100 K–USD 200 K		7.7%, Other
	3.4%, More than USD 200 K		9.0%, Did not specify

**Table 2:** Estimates of ETA and ELR by Days Leading up to the Event.

		Expected Ticket Availability		Expected Lower Rate	
Days Before the Game	Number of Observations	Average (%)	*SD*	Average (%)	*SD*
10	40	53.72	28.88	48.92	29.19
9	80	52.13	24.09	48.13	23.57
8	54	50.33	25.85	46.22	28.49
7	48	46.22	28.47	44.93	28.83
6	71	47.36	28.95	42.53	24.32
5	80	50.08	29.97	43.98	27.65
4	56	48.73	29.81	44.94	28.42
3	60	40.33	22.68	44.31	24.18
2	76	40.81	27.98	38.63	29.21
1	76	37.89	28.15	35.88	29.75

**Table 3:** Estimates of ETA/ELR by Game Demand and Scarcity Framing.

		Expected Ticket Availability		Expected Lower Rate	
Scarcity Framing	Number of Observations	Average (%) ^a^	*SD*	Average (%) ^b^	*SD*
Raw Numbers	208	52.13	26.46	50.38	27.23
Percentages	213	40.63	27.57	38.33	25.87
		**Expected Ticket Availability**		**Expected Lower Rate**	
**Game** **Demand**	**Number of Observations**	**Average (%) ** **^c^**	***SD***	**Average (%) ** **^d^**	***SD***
Low	203	49.50	30.32	46.52	29.24
High	218	43.35	25.58	42.21	25.50

^a^ Main effects result, *p* < 0.001. ^b^ Main effects result, *p* < 0.001. ^c^ Main effects result, *p* < 0.05. ^d^ Main effects result, *p* < 0.10.

## Data Availability

Raw data supporting the conclusions of this article will be made available by the authors, upon request.
